# Effects of foliar selenium spraying on the growth and selenium content and morphology of rice

**DOI:** 10.3389/fpls.2025.1587159

**Published:** 2025-04-25

**Authors:** Wenxia Pei, Mengya Dai, Sheng Shi, Yuan Zhang, Daxia Wu, Cece Qiao, Yafei Sun, Jianfei Wang

**Affiliations:** ^1^ College of Resource and Environment, Anhui Science and Technology University, Fengyang, China; ^2^ ECO−Environment Protection Research Institute, Shanghai Academy of Agricultural Sciences, Shanghai, China

**Keywords:** selenium, rice, selenium distribution, organic se, agronomic traits

## Abstract

Selenium (Se), an essential micronutrient for both plants and humans, plays critical roles in crop metabolism and human physiological functions. However, optimizing Se biofortification strategies to enhance grain Se accumulation while mitigating potential agronomic trade-offs remains a significant challenge. In this study, foliar applications of sodium selenite at concentrations of 0.0075 kg/hm² (FX01) and 0.015 kg/hm² (FX02) were administered during the full heading stage of rice (Oryza sativa L.) to systematically investigate Se uptake, interorgan translocation, and organic Se speciation in grains. Results demonstrated that foliar Se application significantly increased total Se contents and accumulation across rice tissues, with FX02 exhibiting superior enhancement compared to FX01. Specifically, total Se and organic Se contents in rice grains of FX02 were 2.76- and 2.77-fold compared to FX01, respectively. Translocation dynamics revealed that foliar treatment reduced Se transfer rates from leaves to husks and stems, while FX02 markedly improved phloem-mediated Se remobilization from leaves to grains. The Se translocation factor (TF) from leaves to grains increased to 0.71 under FX02, compared to 0.44 in FX01 and 0.60 in CK, indicating enhanced efficiency of Se redistribution under FX02. Spatial partitioning analysis further confirmed reduced Se retention in stems and husks alongside elevated accumulation in leaves under foliar treatments. Notably, Se accumulation in rice grains reached 24% under FX02, significantly higher than CK (15%) and FX01 (14%). Foliar Se application also increased the total organic Se and different organic Se forms contents in grains and altered its composition by reducing the proportion of RNA-bound Se. Temporal analysis revealed that total Se concentrations in rice tissues rose sharply within the first 14 days post-application, followed by a decline in vegetative tissues but a continued increase in grains after 31 days. In addition, grain Se enrichment showed no significant correlation with yield-related agronomic parameters. This study elucidates the dynamic transport-transformation mechanisms of foliar-applied Se in rice, providing a theoretical framework for designing precision Se biofortification strategies that synergistically improve grain nutritional quality and field adaptability.

## 1 Introduction

Selenium (Se) is a vital micronutrient that is crucial for physiological processes in both plants and animals (Chen et al., 2023). First identified in 1817 by Swedish chemist Jons Jacob Berzelius, Se plays a pivotal role in various biological functions (Lanza and Dos Reis, 2021; Zhang and Chu, 2022). Prolonged Se deficiency has been linked to numerous human diseases, including hypertension, hyperlipidemia, and cardiovascular diseases (Lara et al., 2019). Despite the World Health Organization’s (WHO) recommendation of a daily intake of 55 μg/day for adults to maintain health, Se deficiency remains prevalent, affecting over 1 billion individuals across more than 40 countries and regions (Zhang et al., 2019; Hui et al., 2021). Consequently, there is a growing scientific focus on Se supplementation.

Given the absence of endogenous selenium biosynthesis in higher organisms, human Se intake primarily depends on dietary sources. Therefore, there has been increasing interest in strategies aimed at enhancing the Se content of food crops through Se biofortification practice (Roberto et al., 2020; Wang et al., 2021; Zhou et al., 2021). Numerous investigations have delved into Se biofortification across diverse plant species, including potato (Zhou et al., 2019), maize (Wang et al., 2022), wheat (Liang et al., 2020; Xia et al., 2020), soybean (Silva et al., 2023), and rice (Roberto et al., 2018). These initiatives are geared toward combating Se deficiency and enhancing the nutritional profile of staple food crops.

Addressing Se deficiency has drawn significant attention toward Se biofortification practices, encompassing both soil and foliar Se applications, owing to their simplicity and efficacy (Dinh et al., 2019). Soil Se application involves intricate interactions between Se and the soil matrix, where Se is absorbed by plant roots and subsequently transported through the xylem to storage organs, leaves, and ultimately to grains, such as wheat, via the phloem (Li et al., 2008; Ducsay et al., 2016; Gupta and Gupta, 2017; Wang et al., 2019). Conversely, the foliar spray application of Se offers a more readily available route for plant uptake compared to soil application (Zhang et al., 2014). Directly applying Se to plant foliage presents a convenient, cost-effective, safe, and environmentally friendly approach to augment the Se levels in plants. Foliar application promotes swift nutrient absorption, facilitating the translocation of nutrients to other plant tissues.

Following foliar Se application, Se penetrates leaves either through cuticular penetration or the stomatal pathway (Saha et al., 2017). Se is subsequently translocated to edible plant parts, although the degree of re-translocation is contingent upon the plant’s nutritional status and phenological stage (O’Connor et al., 2017). In cereal crops such as wheat, leaf maturity is pivotal in determining whether a leaf acts as a sink or source of Se for grain translocation. Mature leaves have the capability to directly transport Se via the phloem to grains but lack the ability to import Se (Saha et al., 2017). Recent research indicates that foliar Se application during later growth stages may be more efficacious in enhancing the Se contents of plants (Deng et al., 2017; Dinh et al., 2019).

Rice, as a staple food for more than half of the global population and a primary dietary component in China, represents a critical biofortification target for selenium supplementation to alleviate micronutrient malnutrition (Farooq et al., 2019). Current methodologies for selenium agronomic biofortification in Oryza sativa L. bifurcate into genetic enhancement through exploitation of natural Se-accumulating germplasm (Wang et al., 2016) and exogenous selenium application strategies. Investigations into the agronomic biofortification of rice have shown the effectiveness of both soil and foliar Se application in augmenting the Se levels in edible plant parts (Boldrin et al., 2018).Notably, field experiments have shown that when applying 75 kg Se ha^-1^ as selenite [Se(IV)] or selenate [Se(VI)] during the late tillering or full heading stage results in differences in Se distribution coefficients (Deng et al., 2017). Particularly, foliar application Se(VI) at the panicle initiation stage can maximize the proportion of organic Se in polished rice (Farooq et al., 2019). Furthermore, studies have explored the effects of varying concentrations of sodium selenate and sodium selenite sprayed at different growth stages on the Se content in edible plant parts (Marques et al., 2020). These investigations provide valuable insights into optimizing Se biofortification strategies in rice cultivation, with the aim of improving the nutritional quality of rice and addressing Se deficiency in regions where rice consumption is widespread.

However, the existing literature notably lacks comprehensive investigations into the adjuvant-optimized and dose-dependent foliar Se application strategies on grain Se accumulation dynamics in rice. Exploring the effects of foliar-applied Se on rice plants could provide valuable insights into the potential of this technique for Se biofortification programs. Moreover, such research endeavors play a crucial role in addressing the growing concern regarding Se deficiency in regions where rice is a dietary staple.

This study specifically evaluates dose-response relationships in foliar Se application, utilizing adjuvant combinations to enhance phyllospheric absorption during full heading stage, quantifying its dual impact on grain Se enrichment and yield-related parameters. Through a systematic examination of the effects of varying Se concentrations, the objective of this work was to identify the optimal foliar spraying strategy to maximize Se enrichment in rice while simultaneously ensuring its nutritional and agronomic integrity.

## 2 Materials and methods

### 2.1 Experimental design and materials

The field experiment was conducted using a single-factor randomized complete block design (RCBD) from late May to early November 2023 at Danyanghu Farm in Dangtu County (31°21’22”N–31°23’20”N, 118°42’50”E–118°44’48”E). The experiment consisted of 14 experimental plots, each covering 0.333 ha. The treatment structure included the following: a control group (CK) with four replicates, FX01 treatment with four replicates, and FX02 treatment with four replicates. The area has a temperate climate with an average annual temperature of 15.7°C. The terrain primarily features flat topography, with minimal elevation changes not exceeding 3 m. Tidal soil originating from the Yangtze River’s alluvial deposits constitutes the predominant soil type in the experimental site.

The experimental material comprised Oryza sativa ssp. japonica cv. ‘Nanjing 46’, cultivated in plow layer (Ap horizon) soil with the following characteristics: pH 6.41 (1:2.5 H_2_O), organic matter 24.3 g/kg (Walkley-Black method), Available N 114.3 mg/kg (alkaline hydrolysis), available P 49.6 mg/kg (Olsen method), exchangeable K 105.7 mg/kg (NH_4_OAc extraction) and Total Se 0.21 mg/kg (HF-HNO_3_ digestion/HG-AFS), representing marginal Se-deficient soil (<0.4 mg kg^-1^ threshold per GB 15618-2018).

The experiment comprised three treatments: CK, which served as the control with blank water spraying, and FX01 (low Se concentration) and FX02 (high Se concentration), which were Se rich agents with sodium selenite levels of 0.0075 and 0.015 kg/hm^2^, respectively. Furthermore, gibberellin at 0.015 kg/hm^2^, urea at 0.900 kg/hm^2^, sodium glutamate at 0.450 kg/hm^2^, potassium dihydrogen phosphate at 0.750 kg/hm^2^, and sodium dodecylbenzene sulfonate at 0.225 kg/hm^2^ were simultaneously added to each treatment. The mixture was evenly dispersed in 450 kg/hm^2^ of water before spraying, and foliar Se was applied during the full heading stage.

### 2.2 Measurement of soil chemical properties

The foundational agrochemical characteristics of the soil were evaluated according to the methodologies described by Bao (2000). The pH levels were measured with a PB-10 (Sartorius, Germany). The organic matter content was determined volumetrically utilizing the potassium dichromate method. The Available N content was quantified through alkali dissociation diffusion techniques. The available P level was assessed using colorimetric analysis with the molybdenum-antimony method. The exchangeable K content was determined utilizing an FP6410 flame photometer (Xingyi, China).

### 2.3 Measurement of total and organic Se in rice plants

Rice plant samples (grains, husks, leaves, stems and roots) were collected from the field in the maturity stage, and they underwent a series of processing steps, including washing, separation, drying, crushing, and sieving. Subsequently, approximately 0.2500 g of each sample was carefully weighed into a polytetrafluoroethylene digestion tank. Following this, 10 mL of nitric acid and 2 mL of hydrogen peroxide were added (with the inclusion of hydrofluoric acid for rice plant and root parts), initiating a suitable reaction for microwave digestion. Following digestion, the acid was heated to induce evaporation, and 5 mL of 1:1 hydrochloric acid was introduced for reduction. Subsequent heating and acid evaporation led to a decrease in volume, followed by cooling to the desired volume. Microwave digestion atomic fluorescence spectrometry was then employed to determine the Se content of the rice plants. The spectrometric analyses were validated through continuous monitoring of blank samples (n=15) and standard reference materials (NIST SRM 1568b, GBW10045). The method detection limit (MDL) was calculated as 3σ of blank replicates, ensuring signal-to-noise ratios >10:1 for all reported Se concentrations.

The organic Se content of the rice powder was analyzed using a range of methods, including the dialysis bag method (Chen et al., 2002; Zhou, 2007), as well as alkali-soluble protein extraction, polysaccharide extraction, and RNA extraction techniques (Fang et al., 2012). The substances obtained from these methods were extracted and the selenium content was determined using the methods described above.

### 2.4 Determination of the soil plant analysis development value in rice leaves

SPAD measurements were conducted in the field on October 11 and October 28. For each plot, 15 rice hills were randomly selected, with three plants per hill assessed for SPAD readings. The mean SPAD value from these measurements was recorded as the representative SPAD value for each plot.

### 2.5 Statistics and mapping

Statistical analyses were conducted using SPSS 20.0, with one-way analysis of variance (ANOVA) employed to assess significant differences among treatment means. Duncan’s multiple range test was applied at a 0.05 significance level to determine statistical differences. Each data point represents the mean of three replicates. Figures were created in Origin 2024, which also facilitated Pearson correlation analysis.

The translocation factor (TF) was calculated using the formula:


$$\text{TFa}/\text{b}=\frac{\text{Ca}}{\text{Cb}}$$

Where, TFa/b represents the Se translocation factor from rice part “b” to part “a”, as described by Dinh (Dinh et al., 2019). In this formula, Ca and Cb denote the Se concentrations (mg/kg) in rice parts “a” and “b,” respectively.

## 3 Results

### 3.1 Total Se concentrations in rice leaves, stems, roots, and husks following foliar Se spraying

**Figure 1:**
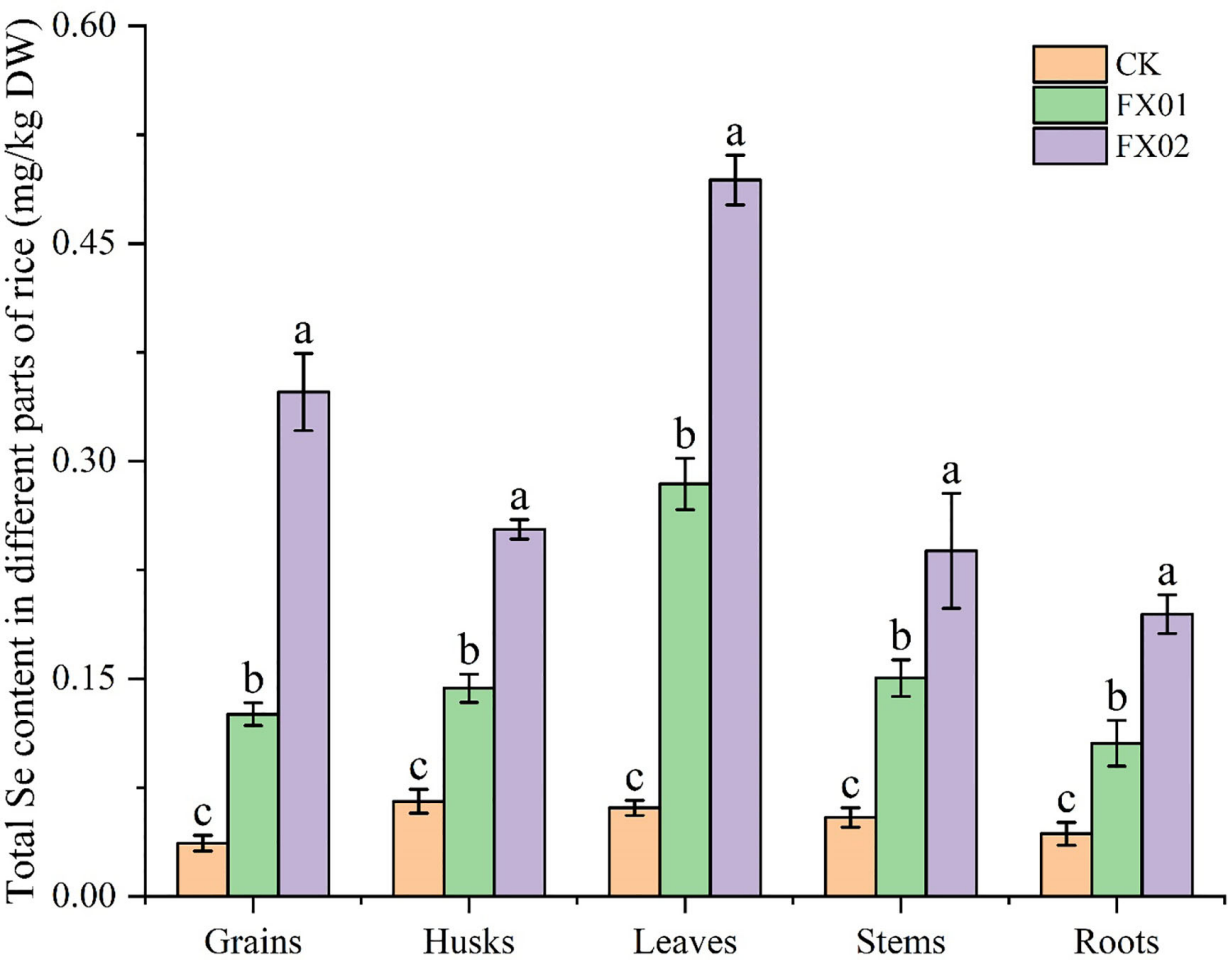
Total selenium (Se) concentrations in different parts of rice (mg/kg). Rice (‘Nanjing 46’) was grown to maturity in the field of Danyanghu Farm. Different tissues (rice grains, husks, leaves, stems, and roots) were harvested and assayed to determine their total Se concentrations. Different lowercase letters above bars indicate significant (*P* < 0.05) differences in the total Se content among the CK (control), FX01 (0.0075 kg/hm^2^ sodium Se), and FX02 (0.015 kg/hm^2^ sodium Se) treatments.

As depicted in **Figure 1**, foliar Se spraying notably augmented the total Se content across various parts of rice, with the enhancement being directly correlated to the quantity of Se applied to the leaves. In comparison to CK, the total Se contents of rice grains, husks, leaves, stems, and roots treated with FX02 increased by 9.48, 3.86, 8.09, 4.38 and 4.50-folds, respectively. Conversely, for rice samples treated with FX01, the total Se contents of rice grains, husks, leaves, stems, and roots, showed increases of 3.42, 2.19, 4.66, 2.77, and 2.44-folds, respectively. In addition, there are significant difference between FX01 and FX02, as illustrated in **Figure 1**.

### 3.2 Translocation factor of Se in rice plant

**Figure 2:**
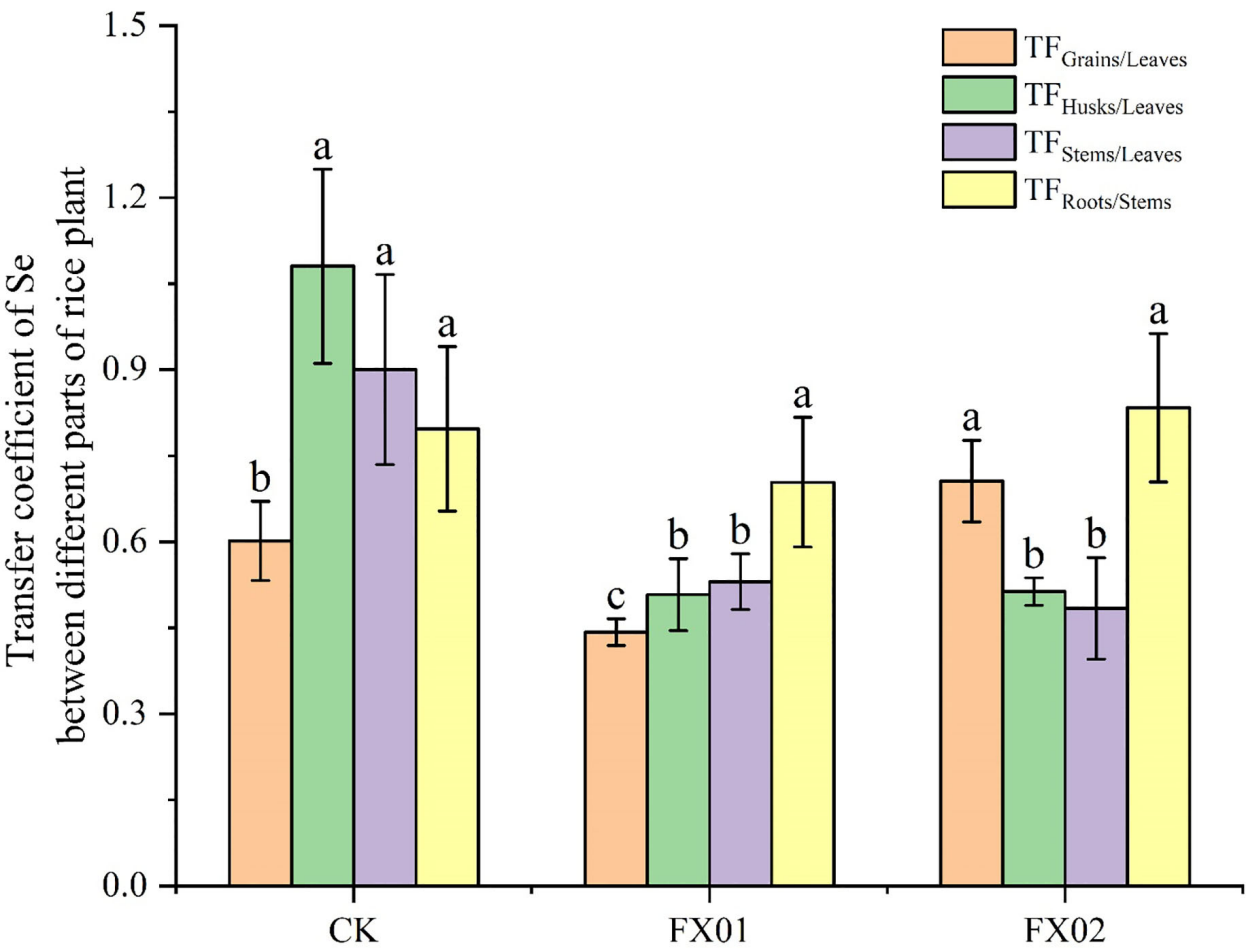
Transfer factor (TF) values of rice under different foliar selenium (Se) treatments. Different lowercase letters above the bars indicate statistically significant differences (*P* < 0.05) in TF values among the CK (control), FX01 (0.0075 kg/hm² sodium selenite), and FX02 (0.015 kg/hm² sodium selenite) treatments. Significant differences (*P* < 0.05) in TF values among different plant parts are also denoted by distinct letters above each bar.

The translocation factor (TF) serves as an indicator of a plant’s capacity to mobilize substances from source to sink (Dinh et al., 2019), with TF values reflecting the efficiency of selenium transfer between plant parts. As shown in **Figure 2**, the Se TF values among different parts of the rice plant displayed distinct patterns across treatments CK, FX01, and FX02. Compared to CK, foliar application of low Se concentrations (FX01) reduced the value of TFGrains/Leaves in rice, whereas high Se concentrations (FX02) significantly increased the TF for leaves to grains movement. Under the CK treatment, TFHusks/Leaves, and TFStems/Leaves were generally higher than those in FX01 and FX02 treatments. However, no significant differences were observed in the values of TFStems/Roots among CK, FX01, and FX02. Notably, the TFHusks/Leaves in the CK group reached approximately 1.2, suggesting a relatively high rate of translocation. These results imply that selenium mobility within the rice plant is influenced by the type of fertilizer treatment, with FX01 and FX02 decreasing selenium transfer to grains compared to CK.

### 3.3 Se accumulation in different parts of rice plant

**Figure 3:**
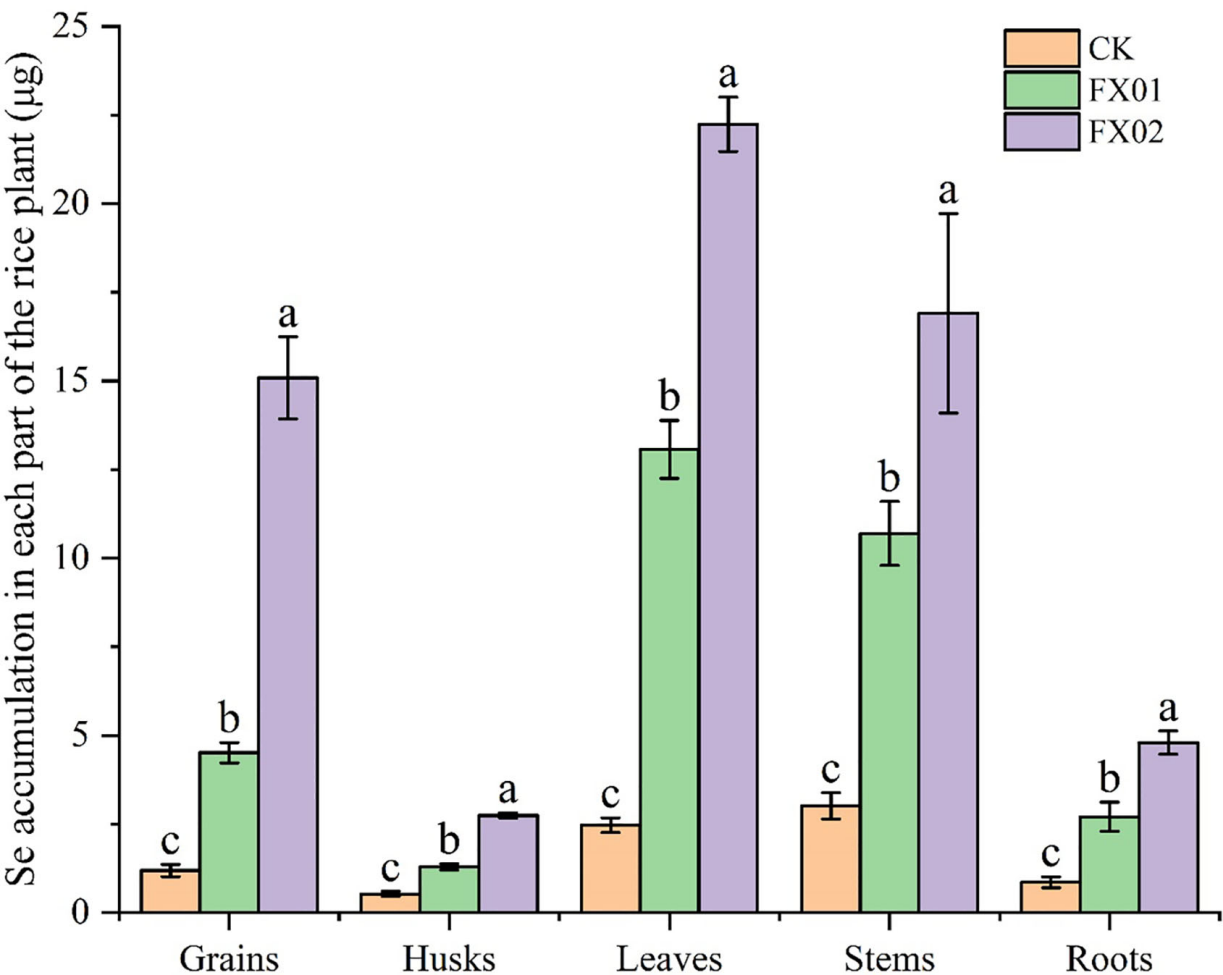
Se accumulation in different parts (grains, husks, leaves, stems and roots) of rice under different foliar Se treatments. Different lowercase letters above bars indicate significant (*P* < 0.05) differences in Se accumulation among the CK (control), FX01 (0.0075 kg/hm^2^ sodium Se), and FX02 (0.015 kg/hm^2^ sodium Se) treatments.

The accumulation of Se in each part of rice is presented in **Figure 3** compared with CK, FX01 and FX02 treatments were significant increased Se accumulation, and was positively correlated with the concentration of Se sprayed on leaves. Across all treatments, Se content was significantly higher in leaves than in other plant parts, with FX02 treatment leading to the highest Se accumulation in leaves at 22.24 μg, was 8.90-folds than that in the leaves of CK (*P* < 0.05). Conversely, husks had the lowest Se accumulation in the CK treatment, was 0.53 μg. In the FX01 and FX02 treatments, Se levels in grains were substantially elevated compared to CK, the accumulation of Se in FX01 and FX02 was 4.52 μg and 15.09 μg, were 3.78 and 12.64-folds than that of CK, respectively. Though the highest Se content remained in the leaves, indicating a strong retention of selenium in vegetative tissues. The variation in selenium accumulation across treatments and plant parts suggests differential absorption and distribution influenced by fertilizer type, with FX02 achieving a more pronounced selenium accumulation in most plant parts compared to FX01 and CK.

### 3.4 Proportion of Se accumulation in each part of the rice plant

**Figure 4:**
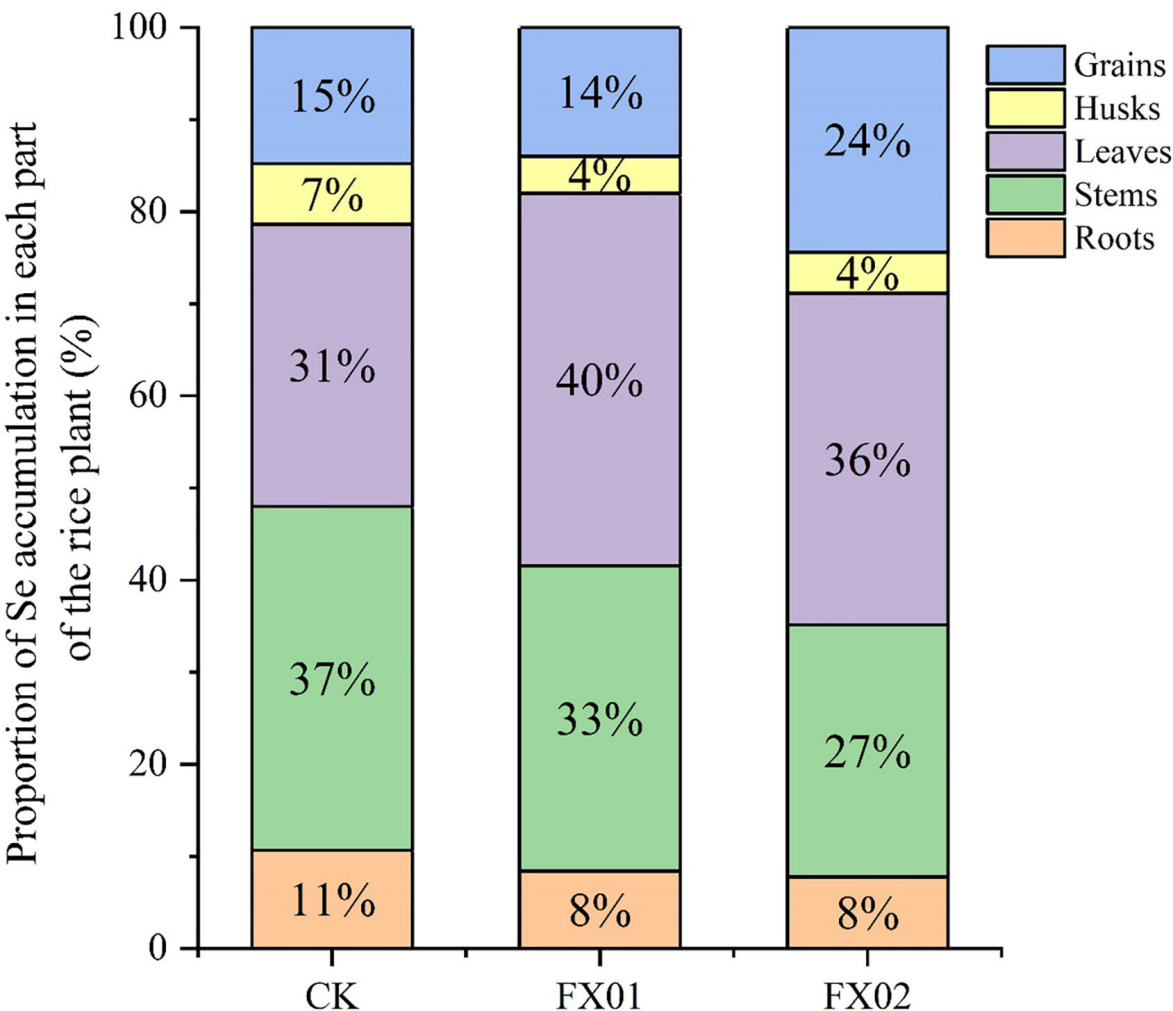
Se distribution in different parts (grains, husks, leaves, stems and roots) of rice under different foliar Se treatments. The Se treatment were CK (control), FX01 (0.0075 kg/hm^2^ sodium Se), and FX02 (0.015 kg/hm^2^ sodium Se) treatments.

**Figure 4** shows the proportional distribution of Se in various plant parts across CK, FX01, and FX02 treatments. In the CK treatment, the stems accounted for the largest proportion of Se accumulation at 37%, followed by leaves at 31%, grains at 15%, roots at 11%, and husks at 7%. Under FX01, the distribution pattern shifted, with leaves representing an increased proportion of 40%, while the proportions in roots and husks slightly decreased, were 8% and 4%, respectively. For FX02, the Se distribution in grains reached 24%, the highest among all treatments, suggesting enhanced selenium translocation to grains compared to CK and FX01. This pattern reflects how FX02 treatment may promote selenium mobilization to edible parts of the rice plant, which could have implications for biofortification purposes.

### 3.5 Concentrations of organic Se and different forms of organic Se in rice grains

**Table 1** illustrates the organic Se and different forms of organic Se content in rice grains. Similar to the findings shown in **Figure 1**, foliar Se spraying demonstrated a significant capability to elevate both the organic and different forms of organic Se contents within rice grains (*P* < 0.05). Compared to CK, the FX01 treatment group exhibited increments of 3.56-folds, while the FX02 treatment group exhibited increases of 8.83-folds. Additionally, it is noteworthy that the organic Se contents of FX02 surpassed those of FX01 by factors of 2.77.

**Table 1:** The content of organic Se and different forms of organic Se in rice grains (μg/kg).

Organic Se forms	CK	FX01	FX02
Organic Se	31.76 ± 1.17c	112.92 ± 9.44b	312.32 ± 20.95a
Alkaline-extracted protein Se	17.57 ± 0.97c	57.31 ± 9.03b	166.14 ± 18.92a
Se-Polysaccharide	3.22 ± 0.40c	10.78 ± 0.76b	31.42 ± 3.85a
RNA Se	0.55 ± 0.03c	0.82 ± 0.01b	1.03 ± 0.02a

Further investigation into the various forms of organic Se content in rice grains revealed that the trends in changes for alkali-extracted protein, polysaccharide, and RNA-bound Se content mirrored those observed for the total and organic Se content in grains (**Figure 1**; **Table 1**). Specifically, the alkali-extracted protein contents in the FX01 and FX02 treatments were 57.31 μg/kg and 166.14 μg/kg, respectively, representing increments of 3.26 and 9.46-folds over that of the CK (17.57 μg/kg), respectively. Similarly, the Se polysaccharide content in CK was found to be 3.22 μg/kg, whereas significant enhancements were observed following foliar Se spraying, with the Se polysaccharide contents of FX01 and FX02 found to be 10.78 μg/kg and 31.42 μg/kg, respectively, constituting increases of 3.35 (FX01) and 9.76 (FX02) folds compared to the CK. For the RNA-bound Se, the contents of RNA-bound Se in FX01 and FX02 were 0.82 and 1.03 μg/kg, were 1.49 and 1.87-folds than those of CK (0.55 μg/kg), respectively.

### 3.6 Effects of spraying Se-enriching agent on the distribution of organic Se content in rice grains

**Figure 5:**
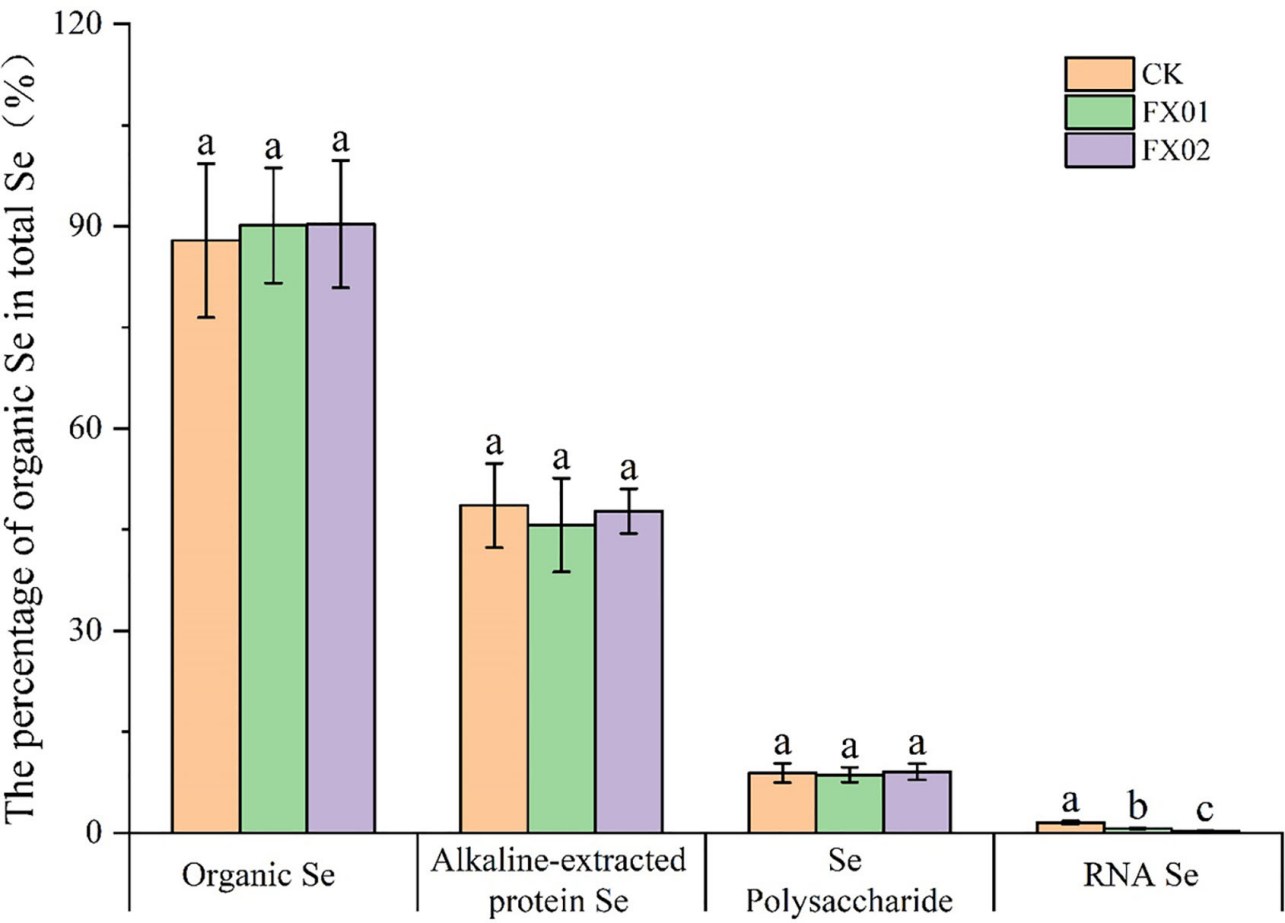
Percentage of organic selenium (Se) in the total Se (%). Different lowercase letters above bars indicate significantly (*P* < 0.05) higher values in the FX01 (0.0075 kg/hm^2^ sodium Se) and FX02 (0.015 kg/hm^2^ sodium Se) treatments compared with the CK (control).

Further analysis involving the calculation of organic Se and its various forms in rice grains, expressed as percentages of the total Se content, is presented in **Figure 5**. In the group of CK, organic Se accounted for 87.89% of the total Se content, with alkali-extracted protein Se comprising 48.59%, polysaccharide Se contributing 8.90%, and RNA Se making up 1.53%. In contrast, in rice grains treated with FX01 and FX02, the organic Se contents rose to 90.13% and 90.32%, respectively. The alkali-extracted protein Se, polysaccharide Se, and RNA Se contents in FX01 accounted for 45.67%, 8.63%, and 0.66%, respectively, while in FX02, these values were 47.74%, 9.07%, and 0.30%, respectively.

Moreover, foliar Se spraying resulted in significant reduced in the ratios of RNA Se in rice grains, was decreased 57.01% (FX01) and 80.49% (FX02) compared with CK (*P* < 0.05). While exerting no discernible impact on the ratios of organic Se, alkali-extracted protein Se and polysaccharide Se. These findings underscore the notable influence of foliar Se application on the distribution of organic Se and RNA Se in rice grains.

### 3.7 Effects of the foliar application of different concentrations of Se fertilizer on the dynamic changes of Se content in various parts of rice plants

**Figure 6:**
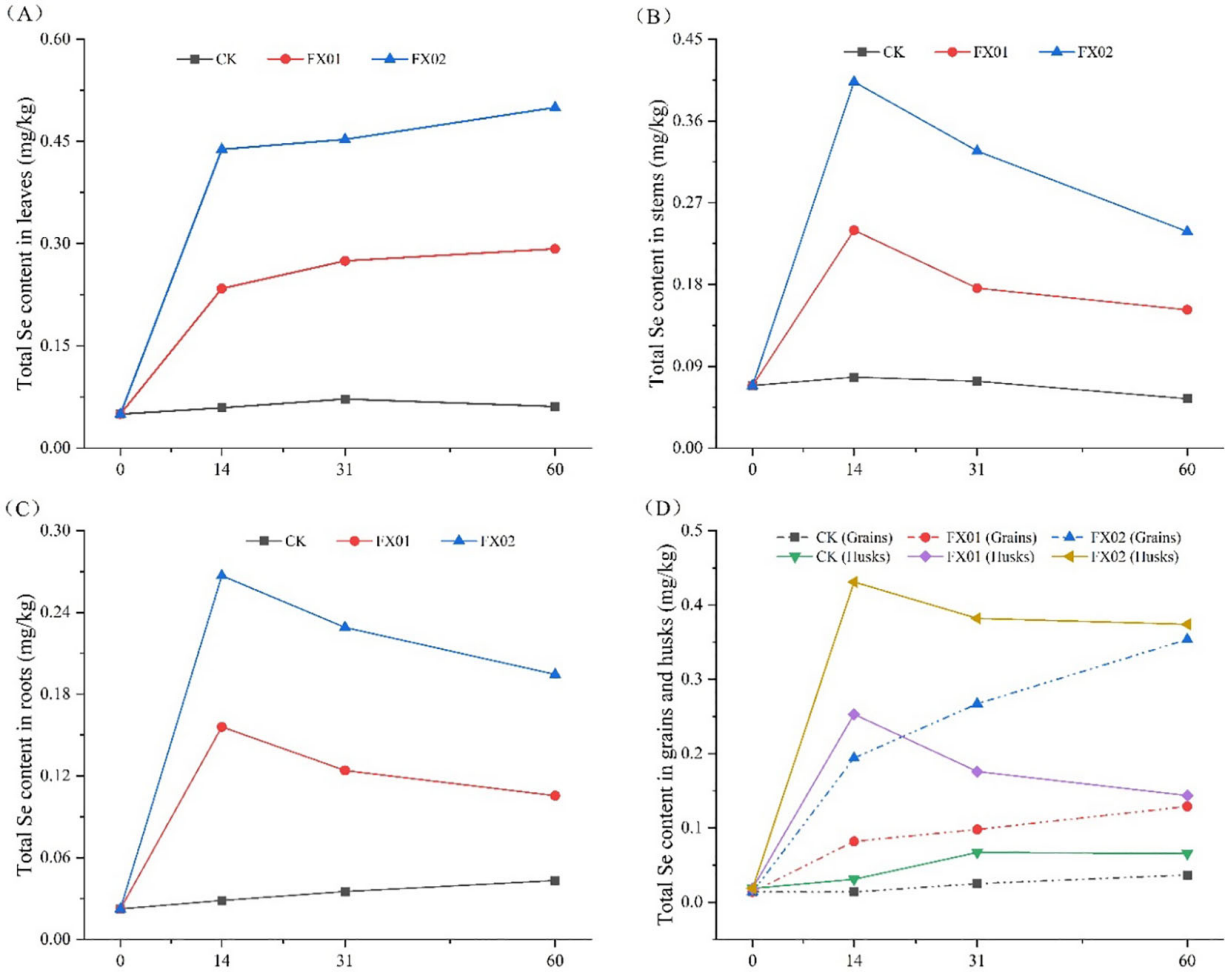
Changes in the selenium (Se) contents of rice leaf blades **(A)**, stems **(B)**, milled rice, rice husks **(D)**, and roots **(C)** at different times after the foliar spraying of different concentrations of Se. Changes in the total Se concentrations of different tissues (leaf blades, stems, rice grains, and roots) at 0, 14, 31, and 60 days after foliar spraying under the CK (control), FX01 (0.0075 kg/hm^2^ sodium Se), and FX02 (0.015 kg/hm^2^ sodium Se) treatments.

To gain deeper insights into the dynamic fluctuations of Se content across various components of rice following foliar Se spraying, measurements were conducted at intervals of 0, 14, 31, and 60 days post-spraying (**Figure 6**). The findings revealed significant increases in the Se contents of leaves, stems, husks, rice grains, and roots compared to CK during the initial 0-14 days post-spraying, with FX02 exhibiting a more pronounced effect (*P* < 0.05). Following this initial phase, a departure from the observed trend occurred after 14 days, wherein the Se contents of leaves and grains continued to exhibit a gradual increase, while the levels of Se in stems, rice husks, and roots exhibited a notable decline. Throughout the entire duration of the experiment, the Se content of each component in the CK group remained relatively stable, showing no discernible alterations over time.

### 3.8 Effects of foliar spraying Se on rice yield and agronomic traits

As shown in **Table 2**, both the FX01 and FX02 treatments yielded significant increases in the biomass of rice stems and whole plants at the mature stage in comparison to CK. Specifically, the dry weights of the whole plants and stems of rice subjected to the FX01 treatment exhibited increments of 17.52% and 28.36%, respectively. Similarly, the FX02 treatment led to increases of 26.78% and 28.10% in the whole plant and stem dry weights, respectively. However, no statistically significant difference was observed between the FX01 and FX02 treatments (*P* < 0.05). The impact of both treatments on rice panicle weight was of particular interest. On average, the dry matter weight of panicles increased by 21.97% following foliar Se spraying. While the FX01 treatment exhibited a 10.53% increase compared to CK, the difference did not reach statistical significance. In contrast, the FX02 treatment significantly elevated the dry matter weight of rice panicles by 33.41% and 20.70% compared to the CK and FX01 treatments, respectively (**Table 2**).

**Table 2:** Effects of different treatments on dry matter accumulation in different parts of rice.

Treatments (g)	Root	Stem	Leaf	Panicle	Plants
CK	19.91a	55.40b	40.51a	92.39b	208.20b
FX01	25.68a	71.11a	45.96a	102.12b	244.68a
FX02	24.68a	70.97a	45.04a	123.26a	263.95a

The different lowercase letters represent different levels of significance.

The SPAD value serves as an indicator of the relative leaf chlorophyll content, which is correlated with the biomass. Further examination of the rice leaf SPAD values revealed that they were unaffected by both the concentration and timing of foliar Se spraying. These findings suggest that foliar Se spraying may not influence the dry weight of rice through alterations in photosynthesis (**Supplementary Figure S1**). Further examination of the biomass distribution ratio of Se across various parts of rice **Supplementary Figure S2**) revealed that rice stems and panicles accounted for a higher proportion of the dry matter of the whole plant, with average distribution ratios of 27.67% and 43.33%, respectively. Conversely, the average distribution ratios of leaf blades and roots were 18.33% and 9.67%, respectively. ANOVA showed that there were no significant differences in the distribution ratio of the biomass among different parts of rice under varying Se treatment levels (*P* < 0.05). This suggests that the application of Se fertilizer has a minimal impact on the distribution of dry matter within rice organs.

The data in **Table 3** indicates the effects of foliar Se application on rice yield and key agronomic traits. Compared to CK, the plant height, number of grains, 1,000-grain weight, and yield in the FX01 and FX02 treatments showed slight decreases, with reductions of 3.53% and 2.09% (plant height), 1.46% and 2.50% (number of grains), 2.59% and 1.26% (1,000-grain weight), and 1.80% and 1.49% (yield), respectively. However, the effective panicle rate exhibited marginal increases of 1.94% and 2.13% (*P* > 0.05) relative to the CK, showing no statistically significant variation between treatments. Interestingly, the effect of FX01 and FX02 on panicle length showed a slight decrease of 3.72% and an increase of 3.72%, respectively, compared to CK. Although there were no significant differences compared to CK, there was a significant difference between the FX01 and FX02 treatments (*P* < 0.05). Overall, compared with CK, foliar Se treatment (FX01 and FX02) did not significantly improve yield or most agronomic traits (*P* < 0.05).

**Table 3:** Effects of foliar Se application on rice yield and agronomic traits.

Treatments	Plant height (cm)	Effective panicle rate (%)	Panicle length (cm)	Number of grains/panicle	1000-grain weight (g)	Yield (kg)/667m^2^
CK	103.83 ± 3.54a	86.00 ± 1.26a	14.33 ± 0.68ab	80.00 ± 1.41a	26.29 ± 1.03a	647.05 ± 9.21a
FX01	100.17 ± 3.19a	87.67 ± 1.63a	13.80 ± 0.51b	78.83 ± 3.37a	25.61 ± 0.49a	635.33 ± 12.21a
FX02	101.67 ± 3.01a	87.73 ± 1.17a	14.87 ± 0.63a	78.00 ± 1.79a	25.96 ± 0.48a	637.33 ± 7.63a

**Figure 7:**
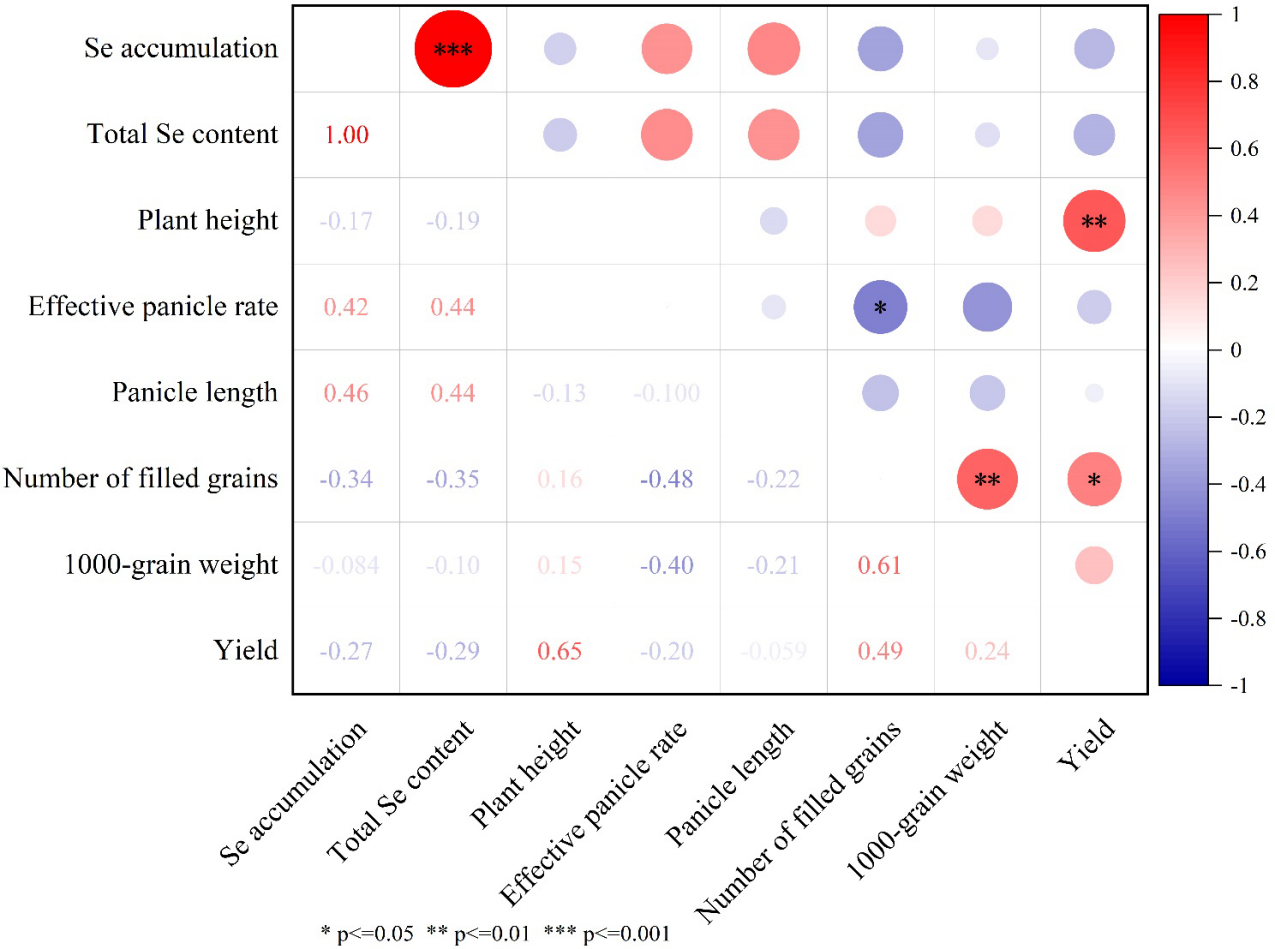
The correlation between total Se content and accumulation in grains, yield, and its main agronomic traits. In the figure, red represents a positive correlation, and blue represents a negative correlation. The deeper the color, the stronger the correlation. * Indicates a correlation at the *P* ≤ 0.05 level, ** indicates a correlation at the *P* ≤ 0.01 level, and *** indicates a correlation at the *P* ≤ 0.001 level.

Comprehensive correlation analysis of Se content, accumulation, yield components, and principal agronomic traits is presented in **Figure 7**. No statistically significant associations were observed between total grain Se content/accumulation and either yield parameters or measured agronomic characteristics. However, a strong positive correlation (*p* < 0.01) emerged between total Se content and accumulation levels. Grain yield demonstrated significant positive correlations with plant height (r=0.65, *p* < 0.01) and filled grains per panicle (r=0.49, *p* < 0.05). Furthermore, 1000-grain weight exhibited a marked positive relationship with filled grain count (r=0.61, *p* < 0.01). Conversely, filled grains per panicle demonstrated a significant inverse correlation with effective panicle rate (r=-0.48, p<0.05).

## 4 Discussion

### 4.1 Total Se concentration and accumulation in different rice organs

It is well known that the concentration and form of Se applied can increase the Se contents and accumulation of rice grains. Currently, soil Se application and foliar Se application are widely used due to their simplicity and practicability (Dinh et al., 2019; Wang et al., 2020). In general, during soil Se application, there are interactions between the soil and Se before the Se is absorbed by plant roots and transported through the xylem to storage parts and leaves and subsequently to grains, such as wheat, via the phloem (Ducsay et al., 2016; Gupta and Gupta, 2017; Wang et al., 2019). Se can also enter the leaves after foliar Se application by penetrating through the cuticle or via the stomatal pathway (Saha et al., 2017). The Se is then transported to the edible parts of the plant, but its re-translocation relies on the nutritional status and phenological stage of the plant (O’Connor et al., 2017). In cereal crops such as wheat, the maturity of leaves determines whether a leaf competes with the grain as a sink of Se or whether it can act as a source for Se translocation to grains. Mature leaves can only transport Se directly via the phloem to grains but cannot import Se (Saha et al., 2017). Thus, both soil and foliar Se application methods may enhance the transport of Se to the edible parts of plants (Boldrin et al., 2018). Recent studies suggest that foliar Se application at later growth stages is more effective for increasing the Se content of plants (Deng et al., 2017; Dinh et al., 2019; Wang et al., 2020). Notably, the safety and regulatory compliance of selenium (Se)-enriched rice are critical considerations in its production and consumption. According to the World Health Organization (WHO) and the Chinese National Standard for Se-enriched rice (GB 22499-2008), the maximum permissible Se concentration in cereals is 0.3 mg/kg. In this study, the Se content in rice grains across different treatments ranged from 0.13 to 0.35 mg/kg. Although the highest recorded value (0.35 mg/kg) marginally exceeds the national threshold, the potential health risk associated with daily consumption remains minimal. For instance, an intake of 200 g of such rice would provide approximately 70 µg of Se, which falls within the safe range defined by the Recommended Dietary Allowance (RDA) and Tolerable Upper Intake Level (UL) (60–400 µg/day) (WS/T 578.3—2017). Furthermore, this level of Se intake could be beneficial in addressing Se deficiencies in populations with inadequate dietary Se intake. These findings underscore the feasibility of foliar Se application as a precise and controllable strategy for producing Se-enriched rice that aligns with both nutritional guidelines and regulatory standards.

In the present research, it was found that compared with CK, spraying an Se-enriched agent onto the leaves could increase the Se contents and accumulations of various parts of rice plants. The Se concentration (0.494 mg/kg) and accumulation (22.239 μg) in the leaf blades, while the total Se content (9.48-folds) and Se accumulation (12.64-folds) increase rate in the rice grains under the FX02 treatment were the highest, proportional to the Se concentration applied via leaf spraying in this treatment (**Figures 1**, **3**). This was consistent with previous findings in crops including wheat (Newman et al., 2019), rice (de Lima Lessa et al., 2020), and soybean (Silva et al., 2023).

The Se contents of different parts of rice plants under different foliar Se spray concentrations and times were further analyzed. Fourteen days after foliar Se application, the Se contents of all parts of rice plants (leaves, stems, grains, husks, and roots) showed significant increases compared to CK (**Figure 4**). The rapid increases in the Se contents of leaves and stems may be attributed to their early exposure to exogenous Se. The increases in the Se contents of grains and husks during the reproductive growth stage may have resulted from the translocation of Se from nutrient-rich tissues to grains. The increase in the Se content of roots may be due to the systemic spread of the Se absorbed by tissues in contact with exogenous Se throughout the plant. Additionally, the leakage of exogenous Se into the soil during foliar Se spraying may have also contributed to the increase in the Se content of rice roots. At 31 and 60 days after foliar Se spraying, while the Se contents of rice leaves and grains continued to increase, the total Se contents of stems, husks, and roots were lower than those at 14 days (**Figure 4**). These results suggest that the Se enrichment effect was most pronounced in all parts of rice plants during the first 14 days after foliar Se spraying, possibly due to the peak growth period of rice grains, leading to Se translocation to the grains from other parts of the plant. This phenomenon may be related to the translocation pattern of Se in different parts of rice plants, and further research is necessary to elucidate the specific mechanisms.

### 4.2 Translocation factor and accumulation proportion

The translocation factor (TF) is a crucial measure for assessing the efficiency with which plants mobilize elements from one part to another (Dinh et al., 2019). In this study, TF values for total selenium (Se) in different rice tissues varied significantly among treatments, particularly between the control (CK), low Se treatment (FX01), and high Se treatment (FX02). The results indicated that FX01 treatment reduced Se translocation from leaves to grains compared to CK, as evidenced by a lower TFGrain/Leaves value. In contrast, the FX02 treatment significantly enhanced Se movement from leaves to grains, suggesting improved Se mobilization to grain tissues at elevated Se levels (**Figure 2**). This increased translocation under FX02 aligns with biofortification objectives aimed at increasing Se content in grains to enhance nutritional value (Di et al., 2023). Additionally, TF values for husks/leaves (TFHusks/Leaves) and stems/leaves (TFStems/Leaves) in CK were generally higher than those observed under FX01 and FX02 treatments, while TF values for stems to roots remained consistent across all treatments. Notably, CK exhibited a TF value of approximately 1.2 for leaves to husks, indicating relatively high Se mobility within nonedible tissues. This suggests that under natural conditions, Se is often distributed away from grains, potentially as a protective mechanism to prevent excessive accumulation in edible parts (Hawrylak-Nowak and Pogorzelec, 2015). However, higher Se concentrations in the FX02 treatment reversed this pattern, facilitating greater Se accumulation in grains.

The Se distribution patterns (**Figure 4**) further corroborate these findings, revealing a distinct shift in Se allocation across treatments. In the CK group, Se predominantly accumulated in stems (37%) and leaves (31%), with only 15% present in grains. The FX01 treatment redistributed Se more toward the leaves (40%) while decreasing the proportions in roots and husks. Notably, the FX02 treatment led to a significant increase in Se content in the grains, reaching 24% of the total Se distribution—surpassing both CK and FX01. This shift indicates that FX02 treatment enhances Se translocation to the edible parts of the plant, a critical aspect for improving grain Se content for biofortification. These findings imply that adjusting Se application rates can influence Se distribution within rice plants. High Se concentrations (FX02) not only boost grain Se levels but also modify the typical distribution pattern, which could be advantageous for producing selenium-enriched rice. By increasing the bioavailability of Se in edible plant parts, foliar Se treatments such as FX02 may present an effective approach for cultivating nutritionally enhanced crops with potential health benefits. These results lay the ground work for optimizing Se fertilization practices aimed at achieving targeted nutrient profiles in staple crops.

### 4.3 Organic Se concentration and percentage in the rice grains

While the transformation of Se species varies slightly among cereal crops, there has been no significant difference observed in Se bioaccessibility across them. The bioavailability of organic Se compounds is typically high, as evidenced by findings that organic Se is readily absorbed and utilized by the human body (Gupta and Gupta, 2017; Molly et al., 2021). In this study, comparative analyses demonstrated that foliar Se application significantly increased the organic Se content in rice grains compared to CK. Following foliar application, organic Se concentrations ranged from 112.92 μg/kg to 312.32 μg/kg, representing a 3.56- to 9.83-fold increase over the control (**Table 1**). Additionally, the proportion of organic Se in rice grains rose slightly, were 90.13% (FX01) and 90.32% (FX02), compared to 87.89% in the control (**Figure 5**). These findings contrast with those reported by Deng (Deng et al., 2017). In this study, Se bioaccessibility was higher than the 65% reported by Fang (Fang et al., 2012). Muleya noted that corn can effectively convert inorganic Se into organic forms, with over 92% of Se in corn existing as organic Se. Independent of the type of exogenous Se applied, organic Se is typically the dominant form found in Se-enriched crops such as mushrooms and peanuts (Zhou et al., 2019; Luo et al., 2021). Variations in Se bioaccessibility percentages may result from differences in Se application methods and the types of crops studied.

This study further examined the effects of foliar Se application on the various forms of organic Se in rice grains. The results indicated that foliar Se treatment significantly increased the levels of alkali-soluble protein Se, polysaccharide Se, and RNA Se in rice grains, showing a positive correlation with the concentration of foliar Se spray (**Table 1**). Notably, these organic Se forms exhibit distinct biological roles, Alkali-soluble protein Se primarily comprises selenoproteins (e.g., thioredoxin reductase) and selenomethionine (SeMet), which are critical for redox homeostasis and thyroid hormone metabolism in humans, with SeMet absorption efficiency exceeding 90% (Fairweather-Tait et al., 2011; Rayman, 2012). Polysaccharide-bound Se enhances immunomodulatory activity (Turo et al., 2021). RNA-bound Se, though less characterized, may influence post-transcriptional regulation through selenouridine incorporation in tRNA, potentially affecting translation fidelity (Lobanov et al., 2009). Interestingly, while foliar Se application notably reduced the proportion of RNA-bound Se, it did not significantly alter the proportions of alkali-soluble protein Se or polysaccharide Se (**Figure 5**). This selective shift suggests preferential allocation of assimilated Se toward functional protein-bound forms, aligning with the metabolic priority of selenoenzyme synthesis in plants (White, 2018). These findings suggest that foliar Se spraying can effectively enhance the biofortification of rice with these nutritionally relevant forms of Se. Given the critical role of Se in human health and the widespread deficiency of this micronutrient in regions such as Asia, where rice is a staple food, these results have important implications for improving Se intake and mitigating associated health risks. Furthermore, the positive correlations observed between the concentration of foliar Se spray and the levels of alkali-soluble protein Se and polysaccharide Se underscore the dose-dependent nature of Se uptake and accumulation in rice grains. This dose-response relationship is pharmacokinetically significant, as SeMet accumulation in grains follows first-order kinetics relative to foliar Se dosage (Li et al., 2010). These findings provide valuable insights for optimizing foliar Se application protocols to achieve targeted organic Se enrichment in rice crops.

### 4.4 Effect of foliar Se spraying on rice yield and agronomic traits

Foliar Se spraying has shown varying effects on the agronomic traits of rice in previous studies. While some research indicates positive impacts on parameters such as grain yield, seed-setting rate, and 1,000-grain weight (Wang et al., 2019), others have found no substantial effect. Consistent with the latter paradigm, our findings revealed minimal alterations in grain yield and core agronomic performance between FX01/FX02 treatments and the CK group. Quantitative analysis of yield components showed non-significant (*P* > 0.05) marginal declines in grains per panicle, 1000-grain weight, and grain yield across the treatments (**Table 3**). These results collectively suggest that foliar Se application at tested concentrations exerted negligible impacts on primary growth parameters in the studied on Nanjing46 during the trial period. While this finding contrasts with some reports of Se-induced yield increases in crops, it corroborates multiple peer-reviewed studies in rice systems. Yan et al. (2021) systematically demonstrated that various selenium fertilizer treatments had no significant effect on yield in three japonica cultivars (Nanjing 9108, Jiahua 1, Wuyunjing 29) under field conditions. Similarly, Boldrin et al. (2013) reported that rice yield remained largely unaffected by the application of either selenate or selenite fertilizers. Furthermore, Shen et al. (2019) specifically documented that foliar NaSeO_3_ application (15 mg/L) induced no yield changes despite enhancing grain Se content. These variations in yield response may be attributed to factors such as rice variety, climatic conditions in the experimental region, and the concentration of selenium applied (Luo et al., 2019). Notably, dose-dependent phytotoxicity has been well documented in cereal crops when foliar Se exceeds critical thresholds. In rice, Bu et al. (2023) observed significantly yield reduction at the high level of Se (25 mg/kg), maybe attributed to Elevated selenium concentrations induce pro-oxidant activity, triggering oxidative stress through excessive ROS generation, which disrupts cellular redox homeostasis and subsequently leads to metabolic dysregulation and biomass reduction in rice plants. Shen et al. (2019) reported that under high Se concentration (1.47 mg/kg), the rice yield was also significant reduction primarily attributed to the grain numbers per panicle and grain filling rate were reduced compared to control and the medium Se concentration.

In the correlation analysis, there was no significant relationship between total Se content and accumulation in rice grains and the yield or its agronomic traits. These results align with previous findings that indicate Se accumulation in rice may not always correlate with improvements in yield or major agronomic traits (de Lima Lessa et al., 2019). However, rice yield exhibited significant positive correlations with plant height (0.65) and the number of filled grains per panicle (0.49) (Wang et al., 2024) (**Figure 6**). The significant positive correlation between 1,000-grain weight and the number of filled grains (0.61) further highlights the interdependence of these traits on rice yield. Interestingly, the negative correlation between the number of filled grains per panicle and the effective panicle rate (-0.48) suggests that the number of filled grains and effective panicle rate may negative correlation. This is consistent with the inversely correlated among effective panicle number per plant, grain number per panicle and grain weight (Kenji et al., 2019).

In conclusion, although foliar Se application (FX01 and FX02) showed slight improvements in some agronomic traits, it did not significantly enhance rice yield under the conditions tested. The lack of significant correlation between selenium content and agronomic traits further suggests that selenium’s role in rice yield enhancement may be complex and dependent on other environmental and agronomic factors. Future research is needed to explore the underlying mechanisms of selenium’s impact on rice growth, with particular attention to its interaction with soil nutrients, water management, and other stress factors.

## 5 Conclusions

In conclusion, this study elucidates the dose-dependent effects of foliar Se application on rice Se content, and morphology. Our systematic analysis demonstrates that foliar Se spraying induces substantial increases in the total and organic Se contents and accumulations of various parts of rice plants post-treatment. These increases were positively correlated with the spray concentration, indicating the effectiveness of foliar application in Se biofortification. Contrary to initial hypotheses, no statistically significant alterations were observed in rice yield and its key components (including effective panicle rate, Panicle length, Number of grains and 1000-grain weight). These results underscore the potential of foliar Se spraying as a promising strategy to address Se deficiency in rice crops. Furthermore, this study contributes valuable insights into the mechanisms governing Se uptake and distribution within rice plants, laying the foundation for optimized Se application protocols in agricultural practices.

## Data Availability

The original contributions presented in the study are included in the article/**Supplementary Material**. Further inquiries can be directed to the corresponding author/s.

## Supplementary material

Supplementary Figure 1 Variation trend of soil plant analysis development (SPAD) values with growth periods under different treatments. The SPAD values were measured on October 11 (15 days after the foliar selenium (Se) application) and October 28 (30 days after Se application). Different lowercase letters indicate significant (*P* < 0.05) differences in the SPAD values among the CK (control), FX01 (0.0075 kg/hm^2^ sodium Se), and FX02 (0.015 kg/hm^2^ sodium Se) treatments.

Supplementary Figure 2 Accumulated dry matter distribution in different parts of rice under different selenium (Se) treatments. Black, blue, green, and red represent the root, stem, leaf blade, and panicle biomass, respectively. CK: control; FX01: 0.0075 kg/hm^2^ sodium Se; FX02: 0.015 kg/hm^2^ sodium Se.

## References

[B1] Bao S. D. (2000). Analysis of soil agrochemistry. 3rd Edn Vol. 49 (Beijing: China Agriculture Press), 56.

[B2] Boldrin P. F. Faquin V. Clemente A. C. S. de Andrade T. Guimarães G. L. R. (2018). Genotypic variation and biofortification with selenium in Brazilian wheat cultivars. J. Environ. Qual. 47, 1371–1379. doi: 10.2134/jeq2018.01.0045 30512055

[B3] Boldrin P. Faquin V. Ramos S. Boldrin K. Ávila F. Guilherme L. (2013). Soil and foliar application of selenium in rice biofortification. J. Food Compos. Anal. 31, 238–244. doi: 10.1016/j.jfca.2013.06.002

[B4] Bu S. Li Y. Li Y. Tan Z. Zhang M. (2023). Characterization of high selenium tolerance in different selenium-accumulating rice varieties. J. Nucl. Agric. Sci. 37, 1681–1689.

[B5] Chen D. Wu H. Shi X. Xu S. Zhang Z. (2023). Editorial: community series in the mechanism of trace elements on regulating immunity in prevention and control of human and animal diseases, volume ii. Front. Immunol. 14. doi: 10.3389/fimmu.2023.1215080 PMC10242182 37287966

[B6] Chen L. C. Yang F. M. Zhang Y. L. Qiu H. U. Gen P. (2002). Selenium analysis of some polished rice in China and effect of biological selenium-enriched fertilizers on level and chemical constitution of selenium in rice grains. Chin. J. Rice Sci. 16, 341–345. doi: 10.1088/1009-1963/11/5/313

[B7] de Lima Lessa J. H. Araujo A. M. Ferreira L. A. Júnior E. C. S. Oliveira C. Corguinha A. P. B.. (2019). Agronomic biofortification of rice (oryza sativa l.) With selenium and its effect on element distributions in biofortified grains. Plant Soil 444, 331–342. doi: 10.1007/s11104-019-04275-8

[B8] de Lima Lessa J. H. Raymundo J. F. Paula B. C. A. Aurélio D. M. F. Mendes A. A. Santiago F. E. M.. (2020). Strategies for applying selenium for biofortification of rice in tropical soils and their effect on element accumulation and distribution in grains. J. Cereal Sci. 96, 103125. doi: 10.1016/j.jcs.2020.103125

[B9] Deng X. Liu K. Li M. Zhang W. Zhao X. Zhao Z.. (2017). Difference of selenium uptake and distribution in the plant and selenium form in the grains of rice with foliar spray of selenite or selenate at different stages. Field Crops Res. 211, 165–171. doi: 10.1016/j.fcr.2017.06.008

[B10] Di X. Qin X. Zhao L. Liang X. Xu Y. Sun Y.. (2023). Selenium distribution, translocation and speciation in wheat (triticum aestivum l.) After foliar spraying selenite and selenate. Food Chem. 400, 134077. doi: 10.1016/j.foodchem.2022.134077 36084597

[B11] Dinh Q. T. Wang M. Tran T. A. T. Zhou F. Wang D. Zhai H.. (2019). Bioavailability of selenium in soil-plant system and a regulatory approach. Crit. Rev. Environ. Sci. Technol. 49, 443–517. doi: 10.1080/10643389.2018.1550987

[B12] Ducsay L. Ložek O. Marček M. Varényiová M. Hozlár P. Lošák T. (2016). Possibility of selenium biofortification of winter wheat grain. Plant Soil Environ. 62, 379–383. doi: 10.17221/324/2016-PSE

[B13] Fairweather-Tait S. J. Bao Y. Broadley M. R. Collings R. Ford D. Hesketh J. E. (2011). Selenium in human health and disease. Antioxidants & Redox Signaling 14 (7), 1337–83. doi: 10.1089/ars.2010.3275 20812787

[B14] Fang J. Zhu H. Fang F. Gong H. P. Zhang X. Zheng R. (2012). Analysis of selenium forms in selenium-enriched rice. Food Res. Dev. 33, 146–150.

[B15] Farooq M. Tang Z. Zeng R. Liang Y. Zhang Y. Zheng T.. (2019). Accumulation, mobilization, and transformation of selenium in rice grain provided with foliar sodium selenite. J. Sci. Food. Agric. 99, 2892–2900. doi: 10.1002/jsfa.9502 30460691

[B16] Gupta M. Gupta S. (2017). An overview of selenium uptake, metabolism, and toxicity in plants. Front. Plant Sci. 7, 2074. doi: 10.3389/fpls.2016.02074 28123395 PMC5225104

[B17] Hawrylak-Nowak M. Pogorzelec (2015). The dual effects of two inorganic selenium forms on the growth, selected physiological parameters and macronutrients accumulation in cucumber plants. Acta Physiol. Plant 37, 1–13. doi: 10.1007/s11738-015-1788-9

[B18] Hui Y. Xuefeng Y. Zengping N. Yun K. S. MiLing L. Tack F. M.. (2021). The beneficial and hazardous effects of selenium on the health of the soil-plant-human system: an overview. J. Hazard. Mater. 422, 126876. doi: 10.1016/J.JHAZMAT.2021.126876 34416699

[B19] Kenji Y. Yoichi M. Fanmiao W. Peng H. Sayaka T. Hirai T.. (2019). Gwas with principal component analysis identifies a gene comprehensively controlling rice architecture. Proc. Natl. Acad. Sci. U. S. A. 116, 21262–21267. doi: 10.1073/pnas.1904964116 31570620 PMC6800328

[B20] Lanza M. G. D. B. Dos Reis A. R. (2021). Roles of selenium in mineral plant nutrition: ros scavenging responses against abiotic stresses. Plant Physiol. Biochem. 164, 27–43. doi: 10.1016/j.plaphy.2021.04.026 33962229

[B21] Lara T. S. de Lima Lessa J. H. de Souza K. R. D. Corguinha A. P. B. Martins F. A. D. Lopes G.. (2019). Selenium biofortification of wheat grain via foliar application and its effect on plant metabolism. J. Food Compos. Anal. 81, 10–18. doi: 10.1016/j.jfca.2019.05.002

[B22] Li H. McGrath S. P. Zhao F. (2008). Selenium uptake, translocation and speciation in wheat supplied with selenate or selenite. New Phytol. 178, 92–102. doi: 10.1111/j.1469-8137.2007.02343.x 18179602

[B23] Li H. F. Lombi E. Stroud J. L. McGrath S. P. Zhao F. J. (2010). Selenium speciation in soil and rice: influence of water management and Se fertilization. J. Agric. Food Chem. 58 (22), 11837–11843. doi: 10.1021/jf1026185 20964343

[B24] Liang Y. Chen Y. Liu D. Cheng J. Zhao G. Fahima T.. (2020). Effects of different selenium application methods on wheat (triticum aestivum l.) Biofortification and nutritional quality. Phyton (Buenos Aires) 89, 423–435. doi: 10.32604/phyton.2020.09339

[B25] Lobanov A. V. Hatfield D. L. Gladyshev V. N. (2009). Eukaryotic selenoproteins and selenoproteomes. Biochim Biophys Acta. 1790, 1424–1428. doi: 10.1016/j.bbagen.2009.05.014 19477234 PMC3471088

[B26] Luo H. He L. Du B. Wang Z. Zheng A. Lai R.. (2019). Foliar application of selenium (se) at heading stage induces regulation of photosynthesis, yield formation, and quality characteristics in fragrant rice. Photosynthetica 57, 1007–1014. doi: 10.32615/ps.2019.114

[B27] Luo L. Zhang J. Zhang K. Wen Q. Ning F. (2021). Peanut selenium distribution, concentration, speciation, and effects on proteins after exogenous selenium biofortification. Food Chem. 354, 129515. doi: 10.1016/J.FOODCHEM.2021.129515 33756318

[B28] Marques A. C. Lidon F. C. Coelho A. Pessoa C. C. Luis I. C. Scotti-Campos P.. (2020). Quantification and tissue localization of selenium in rice (oryza sativa l., Poaceae) grains: a perspective of agronomic biofortification. Plants 9, 1670. doi: 10.3390/plants9121670 33260543 PMC7760205

[B29] Molly M. Young S. D. Vazquez R. S. LIigowe I. S. Broadley M. R. Joy E. J.. (2021). Selenium speciation and bioaccessibility in se-fertilised crops of dietary importance in Malawi. J. Food Compos. Anal. 98, 103841. doi: 10.1016/j.jfca.2021.103841

[B30] Newman R. Waterland N. Moon Y. Tou J. C. (2019). Selenium biofortification of agricultural crops and effects on plant nutrients and bioactive compounds important for human health and disease prevention - a review. Plant Foods Hum. Nutr. (Dordrecht Netherlands) 74, 449–460. doi: 10.1007/s11130-019-00769-z 31522406

[B31] O’Connor K. Hébert F. Powers J. E. Jordan K. S. Lyons E. M. (2017). Leaf morphology explains the disparity between annual bluegrass and creeping bentgrass growth under foliar fertilization. J. Plant Nutr. 41, 596–608. doi: 10.1080/01904167.2017.1406109

[B32] Rayman M. P. (2012). Selenium and human health. Lancet. 379 (9822), 1256–1268. doi: 10.1016/S0140-6736(11)61452-9 22381456

[B33] Roberto D. Chiara F. M. Beatrice F. Maria B. G. Elisabetta B. Ombretta M.. (2018). Selenium biofortification in rice (oryza sativa l.) Sprouting: effects on se yield and nutritional traits with focus on phenolic acid profile. J. Agric. Food. Chem. 66, 4082–4090. doi: 10.1021/acs.jafc.8b00127 29619819

[B34] Roberto D. Luca R. Beatrice F. Simona M. Paolo B. Alessandro D.. (2020). Current knowledge on selenium biofortification to improve the nutraceutical profile of food: a comprehensive review. J. Agric. Food. Chem. 68, 4075–4097. doi: 10.1021/acs.jafc.0c00172 32181658 PMC7997367

[B35] Saha S. Chakraborty M. Padhan D. Saha B. Murmu S. Batabyal K.. (2017). Agronomic biofortification of zinc in rice: influence of cultivars and zinc application methods on grain yield and zinc bioavailability. Field Crops Res. 210, 52–60. doi: 10.1016/j.fcr.2017.05.023

[B36] Shen J. Jiang C. Yan Y. Zu C. (2019). Selenium distribution and translocation in rice (oryza sativa l.) under different naturally seleniferous soils. Sustainability 11, 520. doi: 10.3390/su11020520

[B37] Silva M. A. Sousa G. F. D. Van Opbergen G. A. Z. Van Opbergen G. G. A. Z. Corguinha A. P. B. Bueno J. M. M.. (2023). Foliar application of selenium associated with a multi-nutrient fertilizer in soybean: yield, grain quality, and critical se threshold. Plants 12, 2028. doi: 10.3390/plants12102028 37653945 PMC10221896

[B38] Turo K. J. Spring M. R. Sivakoff F. S. Delgado de la flor Y. A. Gardiner M. M. (2021). Conservation in post-industrial cities: How does vacant land management and landscape configuration influence urban bees? J. Appl. Ecol. 58 (1), 58–69. doi: 10.1111/1365-2664.13773

[B39] Wang M. Ali F. Qi M. Peng Q. Wang M. Bañuelos G. S.. (2021). Insights into uptake, accumulation, and subcellular distribution of selenium among eight wheat (triticum aestivum l.) Cultivars supplied with selenite and selenate. Ecotoxicol. Environ. Saf. 207, 111544. doi: 10.1016/j.ecoenv.2020.111544 33254403

[B40] Wang M. Ali F. Wang M. Dinh Q. T. Liang D. (2020). Understanding boosting selenium accumulation in wheat (triticum aestivum l.) Following foliar selenium application at different stages, forms, and doses. Environ. Sci. pollut. Res. 27, 717–728. doi: 10.1007/s11356-019-06914-0 31808088

[B41] Wang Y. Wang X. Zhai L. Zafar S. Shen C. Zhu S.. (2024). A novel effective panicle number per plant 4 haplotype enhances grain yield by coordinating panicle number and grain number in rice. Crop J. 12, 202–212. doi: 10.1016/j.cj.2023.11.003

[B42] Wang M. Yang W. Zhou F. Du Z. Xue M. Chen T.. (2019). Effect of phosphate and silicate on selenite uptake and phloem-mediated transport in tomato (solanum lycopersicum l.). Environ. Sci. pollut. Res. 26, 20475–20484. doi: 10.1007/s11356-019-04717-x 31102230

[B43] Wang M. Zhou F. Cheng N. Chen P. Ma Y. Zhai H.. (2022). Soil and foliar selenium application: impact on accumulation, speciation, and bioaccessibility of selenium in wheat (triticum aestivum l.). Front. Plant Sci. 13, 988627. doi: 10.3389/fpls.2022.988627 36186067 PMC9516304

[B44] Wang Y. L. Li W. Zhang G. M. (2016). Screening of selenium enriched rice in Heilongjiang province. Heilongjiang Agric. Sci.. 6, 1–3. doi: 10.11942/j.issn1002-2767.2016.06.0001

[B45] White P. J. (2018). Selenium metabolism in plants. Biochim. Biophys. Acta 1862 (11), 2333–2342. doi: 10.1016/j.bbagen.2018.05.006 29751098

[B46] Xia Q. Yang Z. Shui Y. Liu X. Chen J. Khan S.. (2020). Methods of selenium application differentially modulate plant growth, selenium accumulation and speciation, protein, anthocyanins and concentrations of mineral elements in purple-grained wheat. Front. Plant Sci. 11, 1114. doi: 10.3389/fpls.2020.01114 32849686 PMC7396501

[B47] Yan J. Chen X. Zhu T. Zhang Z. Fan J. (2021). Effects of selenium fertilizer application on yield and selenium accumulation characteristics of different japonica rice varieties. Sustainability 13, 10284. doi: 10.3390/su131810284

[B48] Zhang L. Chu C. (2022). Selenium uptake, transport, metabolism, reutilization, and biofortification in rice. Rice 15, 30. doi: 10.1186/s12284-022-00572-6 35701545 PMC9198118

[B49] Zhang M. Tang S. Huang X. Zhang F. Pang Y. Huang Q.. (2014). Selenium uptake, dynamic changes in selenium content and its influence on photosynthesis and chlorophyll fluorescence in rice (oryza sativa l.). Environ. Exp. Bot. 107, 39–45. doi: 10.1016/j.envexpbot.2014.05.005

[B50] Zhang H. Zhao Z. Zhang X. Zhang W. Huang L. Zhang Z.. (2019). Effects of foliar application of selenate and selenite at different growth stages on selenium accumulation and speciation in potato (solanum tuberosum l.). Food Chem. 286, 550–556. doi: 10.1016/j.foodchem.2019.01.185 30827646

[B51] Zhou X. B. (2007). Effect of foliar application of selenite on selenium accumulation and distribution in rice. Acta Pedol. Sin. 44, 73–78.

[B52] Zhou F. Dinh Q. T. Yang W. Wang M. Xue M. Bañuelos G. S.. (2019). Assessment of speciation and *in vitro* bioaccessibility of selenium in se-enriched pleurotus ostreatus and potential health risks. Ecotoxicol. Environ. Saf. 185, 109675. doi: 10.1016/j.ecoenv.2019.109675 31536913

[B53] Zhou F. Peng Q. Wang M. Liu N. Dinh Q. T. Zhai H.. (2021). Influence of processing methods and exogenous selenium species on the content and *in vitro* bioaccessibility of selenium in pleurotus eryngii. Food Chem. 338, 127661. doi: 10.1016/j.foodchem.2020.127661 32882487

