# Supplementary material for: Effects of foliar selenium spraying on the growth and selenium content and morphology of rice
Source: Front Plant Sci. 2025 Apr 25;16:1587159. doi: 10.3389/fpls.2025.1587159 (PMC12061967; doi:10.3389/fpls.2025.1587159)
Supplement: Supplementary Figure 1 — Variation trend of soil plant analysis development (SPAD) values with growth periods under different treatments. The SPAD values were measured on October 11 (15 days after the foliar selenium (Se) application) and October 28 (30 days after Se application). Different lowercase letters indicate significant (P < 0.05) differences in the SPAD values among the CK (control), FX01 (0.0075 kg/hm2 sodium Se), and FX02 (0.015 kg/hm2 sodium Se) treatments. [file Presentation1.pdf]

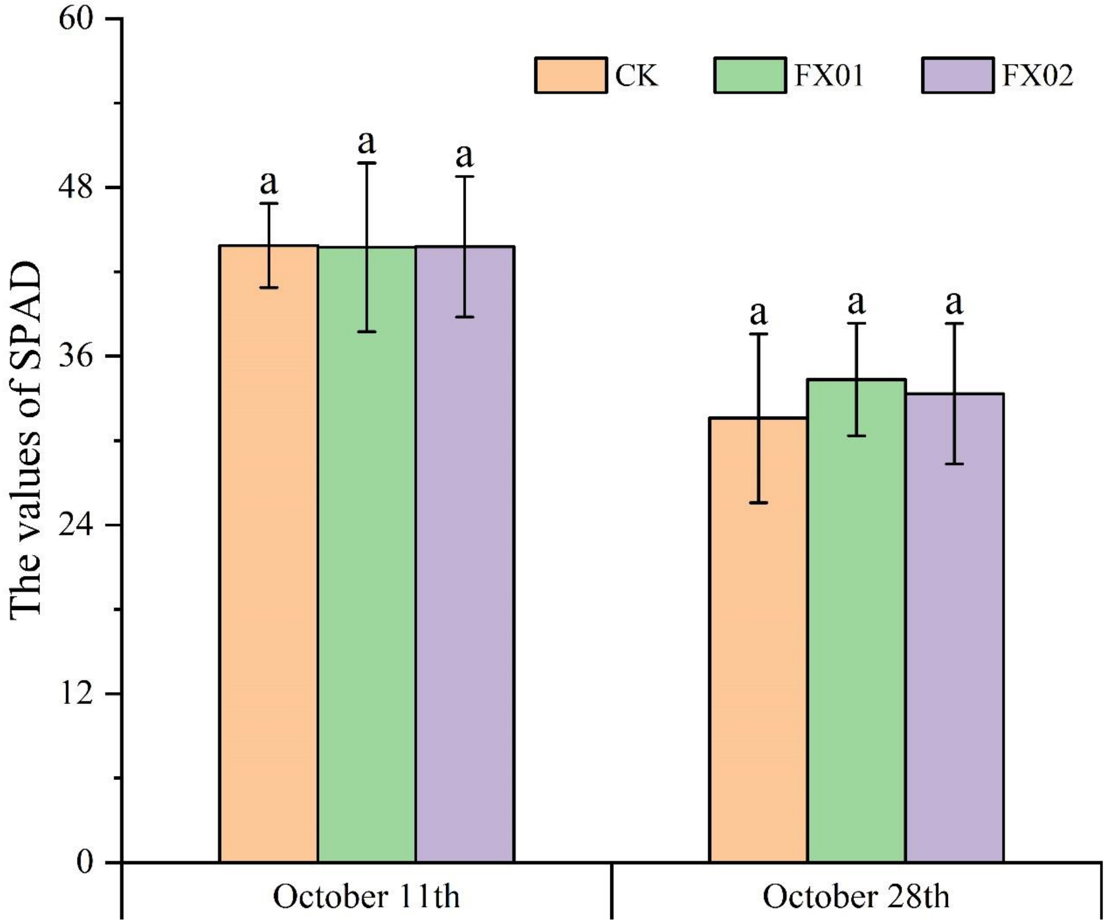

FIGURE S1

Variation trend of soil plant analysis development (SPAD) values with growth periods under different treatments. The SPAD values were measured on October 11 (15 days after the foliar selenium (Se) application) and October 28 (30 days after Se application). Different lowercase letters indicate significant ($P < 0.05$) differences in the SPAD values among the CK (control), FX01 (0.0075 kg/hm^2 sodium Se), and FX02 (0.015 kg/hm^2 sodium Se) treatments.

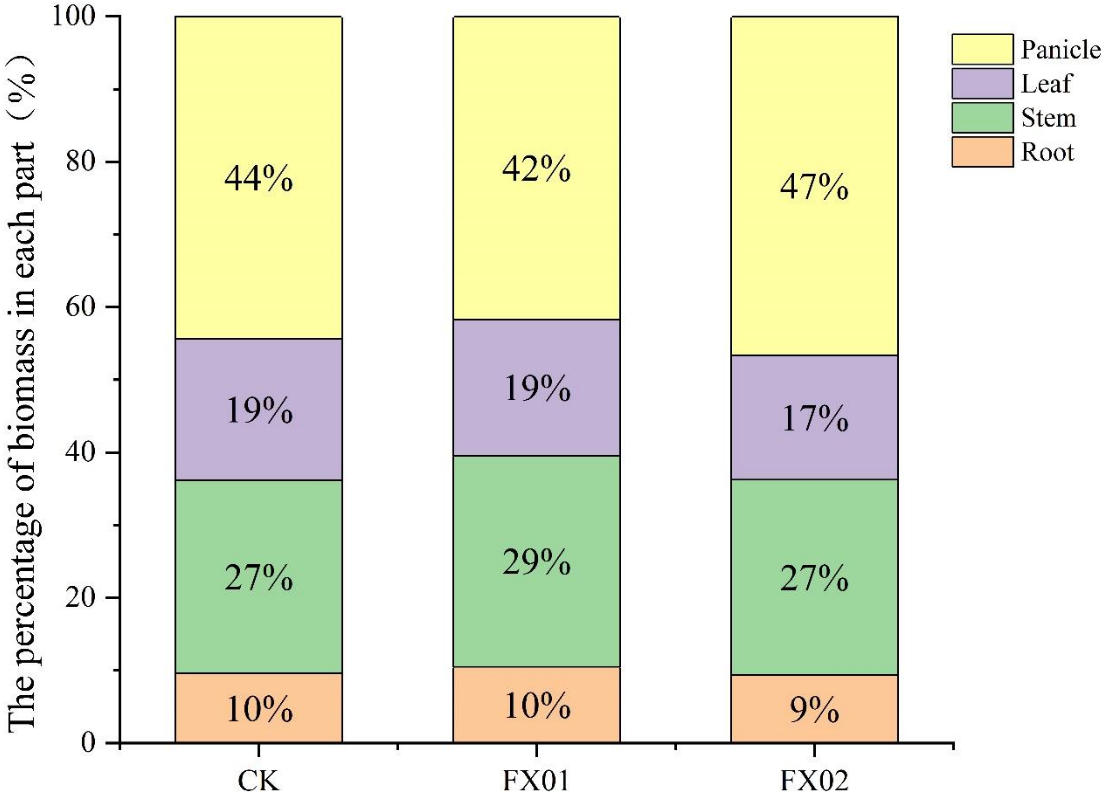

FIGURE S2

Accumulated dry matter distribution in different parts of rice under different selenium (Se) treatments. Black, blue, green, and red represent the root, stem, leaf blade, and panicle biomass, respectively. CK: control; FX01: 0.0075 kg/hm^2 sodium Se; FX02: 0.015 kg/hm^2 sodium Se.
